# Benchmarking Photon-Counting Computed Tomography Angiography Against Invasive Assessment of Coronary Stenosis: Implications for Severely Calcified Coronaries

**DOI:** 10.1016/j.jcmg.2024.11.005

**Published:** 2025-02-11

**Authors:** Rafail A. Kotronias, Giovanni L. de Maria, Cheng Xie, Sheena Thomas, Kenneth Chan, Leonardo Portolan, Jeremy P. Langrish, Jason Walsh, Thomas J. Cahill, Andrew J. Lucking, Jonathan Denton, Robyn Farrall, Caroline Taylor, Nikant Sabharwal, David A. Holdsworth, Thomas Halborg, Stefan Neubauer, Adrian P. Banning, Keith M. Channon, Charalambos Antoniades, the OxAMI and ORFAN Investigators

**Affiliations:** Acute Multidisciplinary Imaging and Interventional Centre, Division of Cardiovascular Medicine, Radcliffe Department of Medicine, University of Oxford, United Kingdom

**Keywords:** 3D QCA, coronary CTA, diagnostic performance, photon-counting

## Abstract

**Background:**

Clinical guidelines do not recommend coronary computed tomographic angiography (CTA) in elderly patients or in the presence of heavy coronary calcification. Photon-counting coronary computed tomographic angiography (PCCTA) introduces ultrahigh in-plane resolution and multienergy imaging, but the ability of this technology to overcome these limitations is unclear.

**Objectives:**

The authors evaluate the ability of PCCTA to quantitatively assess coronary luminal stenosis in the presence and absence of calcification, comparing both the ultrahigh-resolution (UHR)-PCCTA and the multienergy standard-resolution (SR)-PCCTA with the criterion-standard 3-dimensional invasive quantitative coronary angiography (3D QCA).

**Methods:**

The authors included 100 patients who had both PCCTA and invasive coronary angiography (ICA). They comparatively evaluated luminal diameter stenosis with PCCTA and 3D QCA, anatomic disease severity (according to CAD-RADS [Coronary Artery Disease–Reporting and Data System]) and the diagnostic performance of PCCTA in identifying coronary arteries with ≥50% diameter stenosis on 3D QCA requiring invasive hemodynamic severity evaluation and/or revascularization.

**Results:**

The authors analyzed 257 vessels and 343 plaques. UHR-PCCTA luminal evaluation relative to 3D QCA was more precise than SR-PCCTA (median difference: 3% [Q1-Q3: 1%-6%] vs 6% [Q1-Q3: 2%-11%]; *P* < 0.001), particularly in severely calcified arteries (median difference 3% [Q1-Q3: 1%-6%] vs 6% [Q1-Q3: 3%-13%]; *P* = 0.002). Per-vessel agreement for CAD-RADS between UHR-PCCTA and 3D QCA was near-perfect (κ = 0.90 [Q1-Q3: 0.84-0.95]; *P* < 0.001), and it was substantial for SR-PCCTA (κ = 0.63 [Q1-Q3: 0.54-0.71]; *P* < 0.001), especially in severely calcified arteries: κ = 0.90 (Q1-Q3: 0.83-0.97; *P* < 0.001) and κ = 0.67 (Q1-Q3: 0.56-0.77; *P* < 0.001), respectively. Per-vessel diagnostic performance of SR- and UHR-PCCTA was excellent: AUC: 0.94 (95% CI: 0.91-0.98; *P* < 0.001) and 0.99 (95% CI: 0.98-1.00; *P* < 0.001), respectively. UHR-PCCTA diagnostically outperformed SR-PCCTA: ΔAUC: 0.05 (95% CI: 0.01-0.08; *P* = 0.01).

**Conclusions:**

PCCTA compares favorably with ICA for lumen assessment and anatomic disease severity classification in patients presenting with acute coronary syndrome or patients referred for ICA. UHR-PCCTA luminal evaluation is superior to SR-PCCTA, especially in patients with heavy coronary calcification. UHR-PCCTA has excellent diagnostic performance in identifying coronary arteries with ≥50% luminal stenosis on 3D QCA, outperforming standard-resolution imaging.

Coronary computed tomographic angiography (CTA) is the preferred investigation in chronic coronary syndrome patients with low and intermediate clinical likelihood of obstructive coronary artery disease (CAD).^1,2^ Despite the good diagnostic performance of coronary CTA in evaluating the extent, severity, and complexity of CAD,^3–5^ its routine use in patients presenting with high likelihood of CAD, such as non–ST-segment elevation acute coronary syndrome (NSTEACS) presentations, is not supported by contemporary randomized studies,^6–8^ and consequently not recommended by current guidelines.^9^ Luminal evaluation of coronary disease by means of contemporary energy-integrating detector (EID) coronary CTA is limited in the presence of heavily calcified and diffusely diseased coronary arteries. This significantly affects the diagnostic accuracy of EID coronary CTA by reducing its specificity and negative predictive value, leading to nondiagnostic results in up to 10% of patients.^7,8,10^ For that reason, contemporary clinical guidelines do not recommend coronary CTA in the presence of extensive calcification or in patients ≥65 years of age with intermediate to high pretest likelihood of CAD.^1,2^

The recent introduction of photon-counting computed tomography (CT) has generated a range of novel applications beyond the scope of EID CT systems.^11^ Photon-counting coronary computed tomographic angiography (PCCTA) can image coronary arteries at multiple energies and at ultrahigh temporal and spatial resolution (66 ms and 110 µm, respectively) with slice thickness as low as 200 µm.^11^ This high spatial resolution enables visualization of dense structures such as calcified lesions with minimal blooming artefacts, with major impact on visual interpretation of coronary luminal stenoses. Spectrally acquired images allow intrinsic subtraction of calcium and metal artefacts, and they significantly reduce blooming from dense structures otherwise encountered in EID CT imaging, allowing more accurate assessment of the degree of luminal stenosis.^12^ These novel capabilities may be used to improve the precision of coronary evaluation and the diagnostic performance of coronary CTA in patients with extensive, calcified, and complex CAD, extending the clinical indications of noninvasive imaging of the coronaries.

In the present prospective study of patients referred for invasive coronary angiography (ICA), we perform the first comparative evaluation of ultrahigh resolution (UHR) PCCTA and standard-resolution (SR) multi-energy PCCTA relative to ICA for the quantification of coronary luminal stenosis in the presence and absence of coronary calcification.

## Methods

The study took place at the AMIIC (Acute Multidisciplinary Imaging and Interventional Centre) of the University of Oxford from November 2022 to March 2024. We included participants from the OxAMI (Oxford Acute Myocardial Infarction) study and the ORFAN (Oxford Risk Factors and Noninvasive imaging; NCT05169333) study. The OxAMI study prospectively enrolls patients referred for clinically indicated ICA to have a research PCCTA. The ORFAN study screened consecutive patients having ICA after UHR-PCCTA, who were invited for an additional research SR-PCCTA as part of the OxAMI study. Clinical indications for ICA included suspected acute coronary syndrome (ACS) and referral for consideration of revascularization for chronic coronary syndrome refractory to guideline-directed medical therapy. Eighteen participants were referred for ICA following their PCCTA findings. Exclusion criteria for the present study included patients with active malignancy, brittle bronchospastic disease, and estimated glomerular filtration rate (eGFR) <30 mL/min/1.73 m^2^. The OxAMI (11/SC/0397) and the ORFAN studies (15/SC/0545) were both conducted with the approval of the South Central–Oxford C Research Ethics Committee.

### Photon-Counting Coronary CTA

PCCTA was performed at AMIIC. In preparation for the scans, participants had 800 µg of sublingual glyceryl trinitrate and intravenous metoprolol up to a maximum of 40 mg aiming for a target heart rate of <60 beats/min. PCCTA imaging was performed with a first-generation system (NAEOTOM Alpha with software VA50 SP1, Siemens Healthineers). The PCCTA imaging protocol included both SR and UHR acquisitions. The combined protocol was performed in 67 participants. SR-PCCTA was not performed in 11 participants. Six participants declined the additional scan, 1 participant was found on the first scan to have overt malignancy, for 1 participant the scan was aborted because of a scan planning error, and for 3 participants it was not feasible to perform the second scan. UHR-PCCTA was not performed in 22 participants. In 21 participants the estimated radiation exposure would have exceeded the protocol allowance, and 1 participant withdrew consent for the UHR-PCCTA.

Quantum Plus multienergy acquisitions were acquired with prospective gating (Flash protocol) guided by the participant’s heart rate and rhythm, with a gantry rotation time of 0.25 seconds in the axial plane at a slice thickness of 0.4 mm. UHR acquisitions were retrospectively gated (Flex or Spiral protocols) with a gantry rotation time of 0.25 seconds in the axial plane at a slice thickness of 0.2 mm. Tube voltage was set at 140 kV for the SR-PCCTA and 120 kV for the UHR-PCCTA, and tube current was automatically adjusted to the chosen image quality level of each acquisition with the use of combined applications to reduce exposure (CARE Dose4D, Siemens Healthineers).

SR-PCCTA was performed with 70 mL intravenous iodinated contrast media (Omnipaque 350 mg I/mL) (GE HealthCare) followed by a saline-solution chaser of 50 mL with a flow rate of 4.5 mL/s and acquired in Flash mode with a CARE keV image quality level of 89 and electrocardiogram triggering between 50% and 80% of the RR interval. UHR-PCCTA was performed 3 minutes after the SR-PCCTA with 80 mL intravenously administered iodinated contrast media (Omnipaque 350 mg I/mL) followed by a saline-solution chaser of 50 mL with a flow rate of 4.5 mL/s and acquired in Flex or Spiral mode with a CARE keV image quality level of 69 and electrocardiogram-triggered window set between 70% and 80% of the RR interval. Systolic phase imaging was performed when a participant’s heart rate was >70 beats/min. Injection rate was maintained with the use of a dual-syringe injector (Bracco CT Express contrast injection). The field of view of different acquisitions was kept constant. SR-PCCTA acquisitions were reconstructed at different energies (67 keV, 100 keV) with the use of the Quantum PURE Lumen (Siemens Healthineers) mode (a postprocessing analysis removing calcium with a digital subtraction algorithm applied to the 67 keV virtual monochromatic imaging data) for measurement of coronary stenosis. The SR-PCCTA images (acquired at 0.4 mm slice thickness) were reconstructed as a vascular kernel (Bv40 Q4) for visual inspection and a quantitative kernel (QR44 Q4) for quantification. UHR-PCCTA acquisitions were performed using 0.2 mm slice thickness at blended energies and reconstructed with a Bv60 Q4 kernel.

### Invasive Coronary Angiography

ICA was performed either in the AMIIC catheterization laboratory or in the Oxford Heart Centre of the John Radcliffe Hospital with standard diagnostic/guide catheters following contemporary diagnostic coronary angiography practice guidelines. All participants received intracoronary glyceryl trinitrate during ICA, and the operators ensured that suitable orthogonal views of each coronary artery were obtained to enable off-line quantitative coronary angiography.

### Coronary Stenosis Evaluation

ICAs were analyzed off-line with the use of 3-dimensional quantitative coronary angiography (3D QCA) (QAngio XA 3D Suite, Medis Medical Imaging Systems) in the AMIIC core laboratory by personnel (J.W. and L.P.) blinded to the coronary CTA analyses and clinical information. This analysis determined the number of stenoses per AHA coronary segment to be expected in the corresponding PCCTA segment. Where a stenosis involved >1 coronary segment, this was noted to ensure that corresponding stenoses were evaluated. We evaluated by 3D QCA minimum luminal diameter (mm) and percentage diameter stenosis (%) in vessels with a reference diameter of ≥2 mm. Angiography-derived coronary physiology analyses (Medis QFR, Medis Medical Imaging Systems) also were performed to define diffusely and focally diseased coronary arteries according to quantitative flow reserve–based pressure pull back gradient (QFR PPG).^13^ A QFR PPG index of <0.66 indicated diffuse pattern disease, and QFR PPG ≥0.66 indicated focal pattern disease.

PCCTA evaluation was performed on syngo.via (version VB60, Siemens Healthineers) within the AMIIC core laboratory, by 2 readers (R.A.K., with BSCI CT L2 accreditation and 4 years of experience; and C.X., with SCCT L3 accreditation and 8 years of experience). Equivocal cases were evaluated by the 2 readers together and if consensus was not reached, a third reader (C.A.) with >15 years of experience performed the adjudication. All readers were blinded to both the 3D QCA results and any other clinical information. Luminal evaluation in the SR-PCCTA images was performed at 67 keV, which corresponds to 120 kVp acquisition on conventional EIDs.^14^ Stenoses of participants who had both SR- and UHR-PCCTA, were analyzed separately 14 days apart. Pure calcium scoring was used to quantify coronary calcium score (CCS) from contrast PCCTA images, allowing classification of patient’s coronary artery into mildly (<100 AU), moderately (100-300 AU), and severely (>300 AU) calcified. At an individual lesion level, severe calcification was defined as the presence of calcium that encompasses more than 50% of the cross-sectional area of the vessel at any location, as previously recommended.^5^ Severely calcific stenoses were analyzed at 67 keV, 100 keV (not recommended by Siemens Healthineers), and with a calcium subtraction mode known as Quantum PURE Lumen applied to the 67 keV virtual monochromatic imaging data. Plaque- and vessel-level anatomic disease severity was categorized according to the CAD-RADS (Coronary Artery Disease–Reporting and Data System) luminal stenosis classification (Supplemental Table 1).^15^

### Statistical Analysis

Normality assumption was evaluated graphically and statistically with Kolmogorov-Smirnov testing. Data are presented accordingly as mean ± SD or median (Q1-Q3). Relationships of luminal evaluation metrics between modalities were graphically presented and statistically evaluated with the use of Spearman ρ and intraclass correlation coefficient with accompanying 95% CIs. To further evaluate agreement between modalities, Bland-Altman plots with bias estimates are provided. To statistically evaluate precision, mean differences between UHR-PCCTA and SR-PCCTA relative to 3D QCA were compared by means of Levene and Mann-Whitney U tests. Finally, Cohen’s κ statistic is used to evaluate the intermodality agreement for plaque- and vessel-level disease severity. The diagnostic accuracy of SR-PCCTA relative to UHR-PCCTA, for the identification of arteries requiring invasive hemodynamic severity evaluation and/or revascularization (diameter stenosis ≥50% by 3D QCA) is measured as the area under the receiver operating-characteristic curve (AUC). This outcome is key to develop PCCTA as a modality that can guide treatment decision making and revascularization planning. Setting the cutoff at ≥50% diameter stenosis aligns with the luminal diameter stenosis threshold of interventional clinical trials exploring treatment decision making based on ICA in patients with CAD.^16,17^ Values of *P* < 0.05 were considered to indicate statistical significance. All *P* values are 2-sided, and the 95% CIs are also presented. Statistical analyses are performed with the use of SPSS (version 29, IBM Corp).

## Results

### Population

A total of 100 participants were included in this comparative analysis of PCCTA and ICA. The study flow diagram is presented in Supplemental Figure 1. The cohort is representative of contemporary acute and chronic coronary syndrome populations, with the majority being participants with ACS presentations with 2 or more risk factors for atherosclerotic coronary disease (Table 1).

Participants’ heart rates during SR-PPCTA and UHR-PCCTA acquisitions were 59 ± 7 beats/min and 59 ± 6 beats/min, respectively. The median ionizing radiation exposures (dose-length product) for SR- and UHR-PCCTA scans were 101 mGy · cm (Q1-Q3: 78-132 mGy · cm) and 269 mGy · cm (Q1-Q3: 226-352 mGy · cm), respectively. The median effective radiation doses were 2.8 mSv (Q1-Q3: 2.2-3.7 mSv) and 7.5 mSv (Q1-Q3: 6.3-9.9 mSv) (0.028 mSv/mGy · cm conversion factor) for SR- and UHR-PCCTA, respectively. Among the 67 participants who had both SR- and an UHR-PCCTA, the latter amounted to a 2.7-fold (Q1-Q3: 2- to 3.5-fold) increase in radiation exposure compared with SR-PCCTA.

### Luminal Characterization

**Figure 1:**
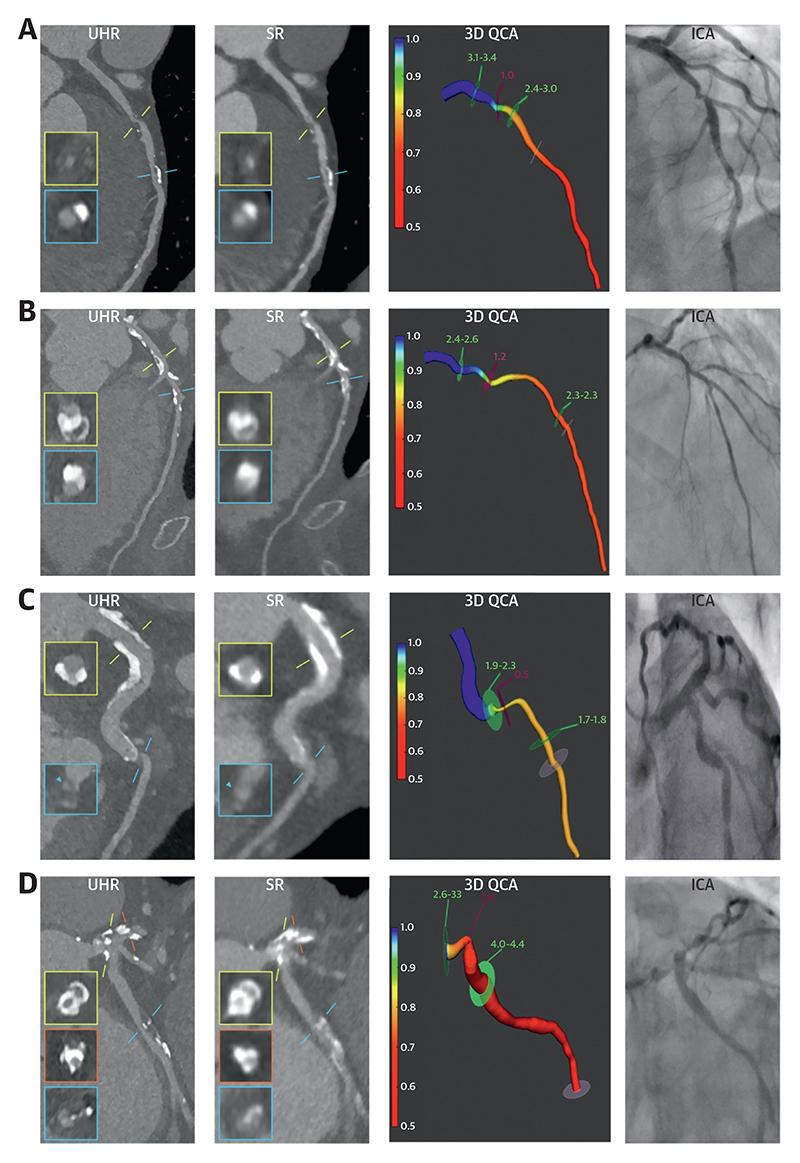
Illustrative Examples of Multimodality Imaging With UHR-PCCTA, SR-PCCTA, and 3D QCA From ICA (A) Mixed and calcified plaques in the mid-LAD causing moderate and mild stenosis, respectively (LAD calcium score: 297 AU; patient calcium score: 476 AU). (B) Diffusely diseased LAD with a calcified plaque in the mid-vessel causing moderate stenosis (LAD calcium score: 1,231 AU; patient calcium score: 3,164 AU). (C) Focal noncalcified plaque in the ostium of the OM1 branch causing severe stenosis that is visualized with UHR-PCCTA but not with SR-PCCTA (LCx calcium score: 61 AU; patient calcium score: 707 AU). (D) Calcified plaques in the distal LM extending into the ostial LAD and LCx arteries causing severe stenoses, and a mixed plaque in the proximal LCx causing moderate stenosis (LM calcium score: 658 AU; LAD calcium score: 649 AU; LCx calcium score: 71 AU; patient calcium score: 1,533 AU). 3D = 3-dimensional; ICA = invasive coronary angiography; LAD = left anterior descending artery; LCx = left circumflex artery; LM = left main coronary artery; OM = obtuse marginal; PCCTA = photon-counting coronary computed tomographic angiography; QCA = quantitative coronary angiography; SR = standard resolution; UHR = ultrahigh resolution.

We analyzed 257 vessels (Central Illustration A, Figure 1, Supplemental Methods), identifying 349 plaques (3 per patient).

A total of 343 plaques were analyzed, as 3D QCA was not possible in 6 stenoses because of extreme fore-shortening of available angiographic projections or aorto-ostial stenoses precluding accurate 3D QCA evaluation. Stenoses with varying degree of calcification and extent of disease were included (Tables 2 and 3).

**Figure 2:**
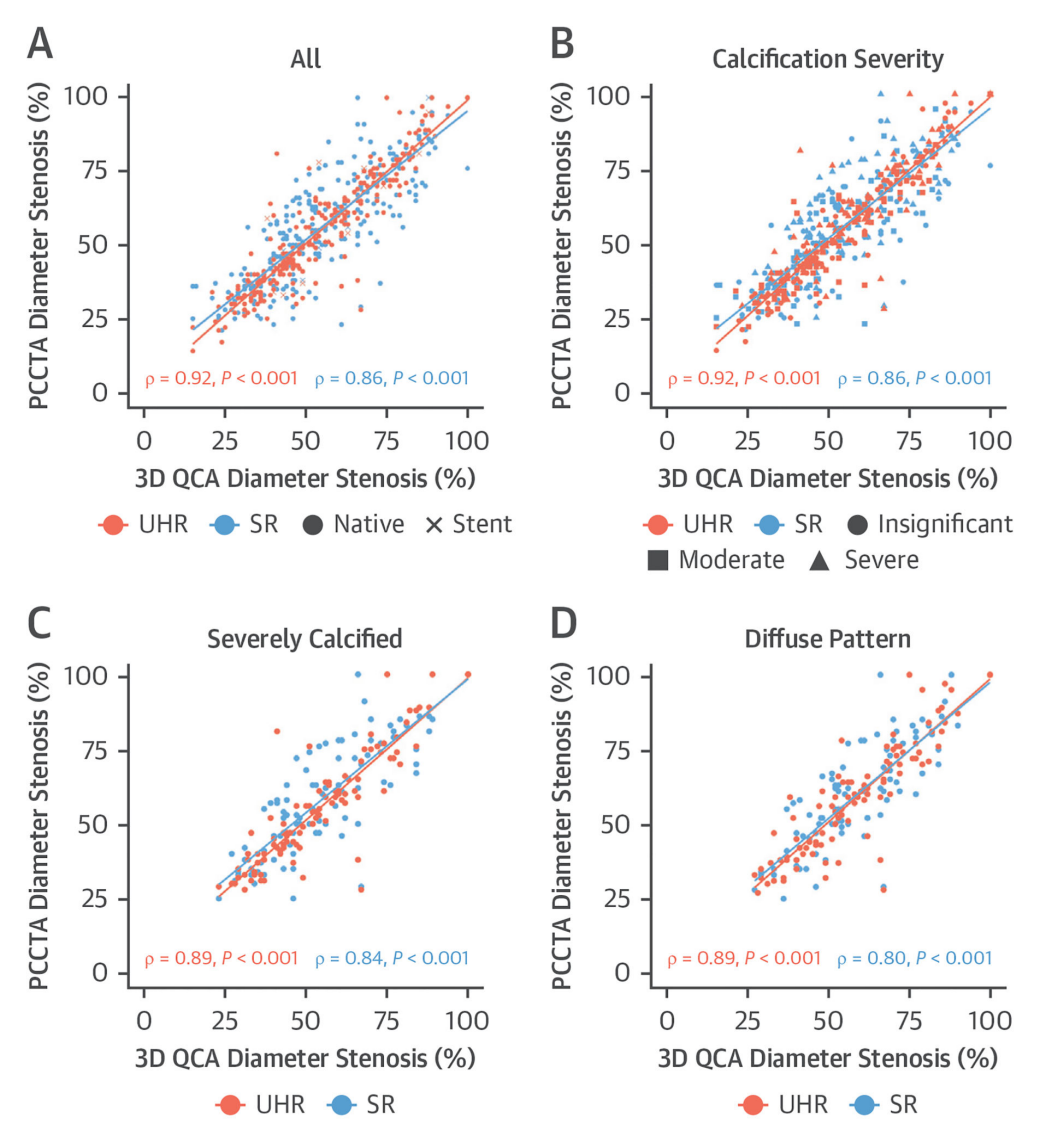
Correlations of UHR- and SR-PCCTA Diameter Stenosis (%) Relative to 3D QCA (A) Across all plaques highlighting native and stented segments; (B) across all plaques highlighting different degrees of calcification; (C) plaques in severely calcified arteries; and (D) plaques in diffusely diseased arteries. Abbreviations as in Figure 1.

**Figure 3:**
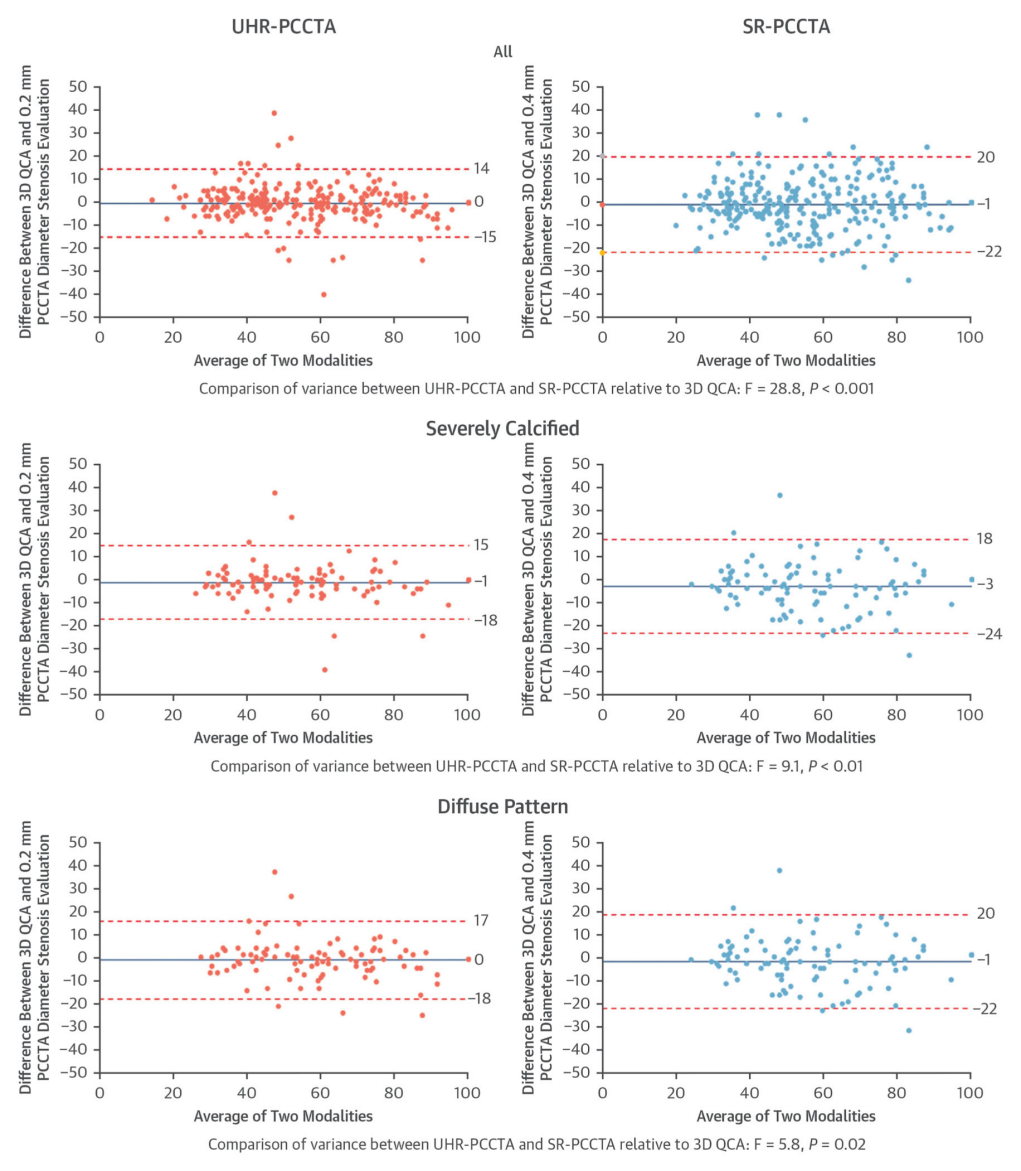
Bland-Altman Plots of UHR-PCCTA and SR-PCCTA Diameter Stenosis (%) Relative to 3D QCA for All Plaques, Severely Calcified Plaques, and Plaques in Diffusely Diseased Arteries Abbreviations as in Figure 1.

**Figure 4:**
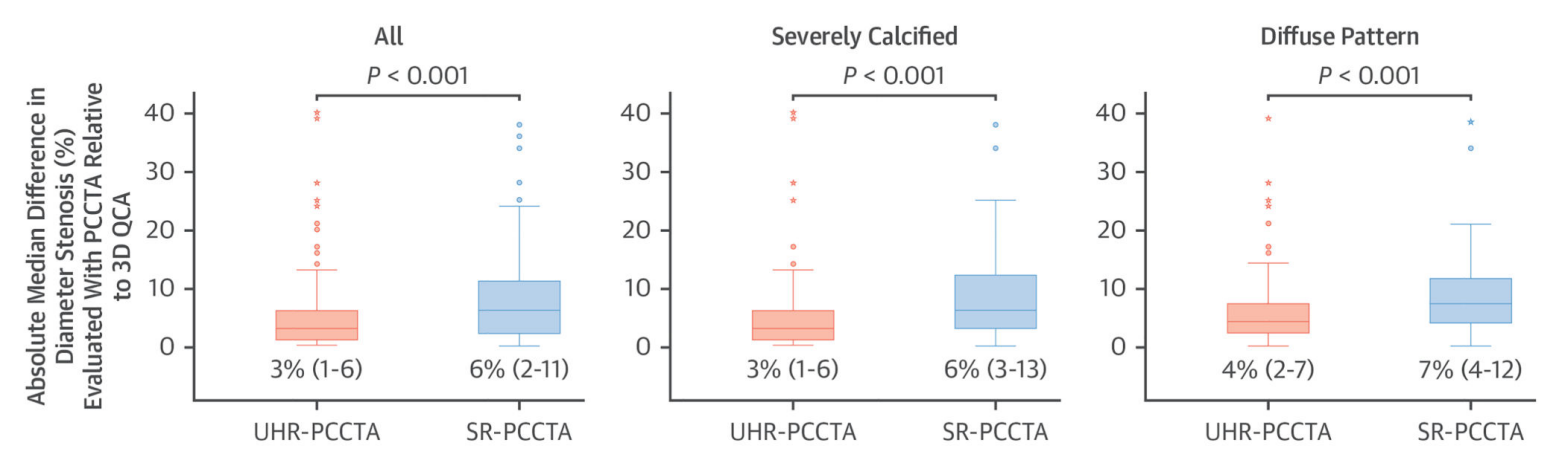
Box Plots of the Absolute Median Difference in Diameter Stenosis (%) of UHR-PCCTA and SR-PCCTA Relative to 3D QCA, Demonstrating Higher Precision of UHR-PCCTA Compared With SR-PCCTA Boxes are median (Q1-Q3). Abbreviations as in Figure 1.

Both SR-PCCTA and UHR-PCCTA luminal evaluation by percentage diameter stenosis and minimum luminal diameter were very strongly correlated and in excellent agreement with 3D QCA evaluation (Table 4, Figure 2, Supplemental Figure 2). The very strong correlations and excellent agreement were also observed in severely calcific, nonseverely calcific, diffuse, and focal stenoses, except for a strong correlation and substantial agreement in minimum luminal diameter of diffuse stenoses between SR-PCCTA and 3D QCA (Table 4, Figure 2, Supplemental Figure 2). Bland-Altman plot analyses (Figure 3, Supplemental Figure 3) demonstrate the precision of luminal evaluation with both SR-PCCTA and UHR-PCCTA, with no evidence of systematic bias. Luminal evaluation with UHR-PCCTA was more precise than with SR-PCCTA (absolute median difference: 3% [Q1-Q3: 1%-6%] vs 6% [Q1-Q3: 2%-11%]; *P* < 0.001), especially in severely calcified (absolute median difference: 3% [Q1-Q3: 1%-6%] vs 6% [Q1-Q3: 3%-13%]; *P* = 0.002) and diffusely diseased arteries (absolute median difference: 4% [Q1-Q3: 2%-7%] vs 7% [Q1-Q3: 4%-12%]; *P* = 0.01) (Figure 4, Supplemental Figure 4).

### Severely Calcific Stenosis Luminal Evaluation

A total of 78 severely calcific stenoses were imaged with both SR-PCCTA and UHR-PCCTA. SR-PCCTA scans were evaluated at 67 keV, 100 keV, and Quantum PURE Lumen reconstructions (Table 5, Supplemental Figure 5). Percentage diameter stenosis and minimum luminal diameter evaluated at 100 keV and Quantum PURE Lumen mode were strongly correlated and in substantial agreement with 3D QCA. SR-PCCTA luminal evaluations at 67 keV and UHR were very strongly correlated and in excellent agreement with 3D QCA (Table 5). Comparatively, luminal evaluation of severely calcified stenoses was less precise with Quantum PURE Lumen algorithm than SR- or UHR-PCCTA monoenergetic image evaluation. From a technical perspective, the current internal subtraction algorithm subtracts contrast from the lumen alongside calcification, resulting in the observed overestimation of the stenoses.

### Comparative Evaluation of Anatomic Disease Severity According to PCCTA and 3D QCA

**Figure 5:**
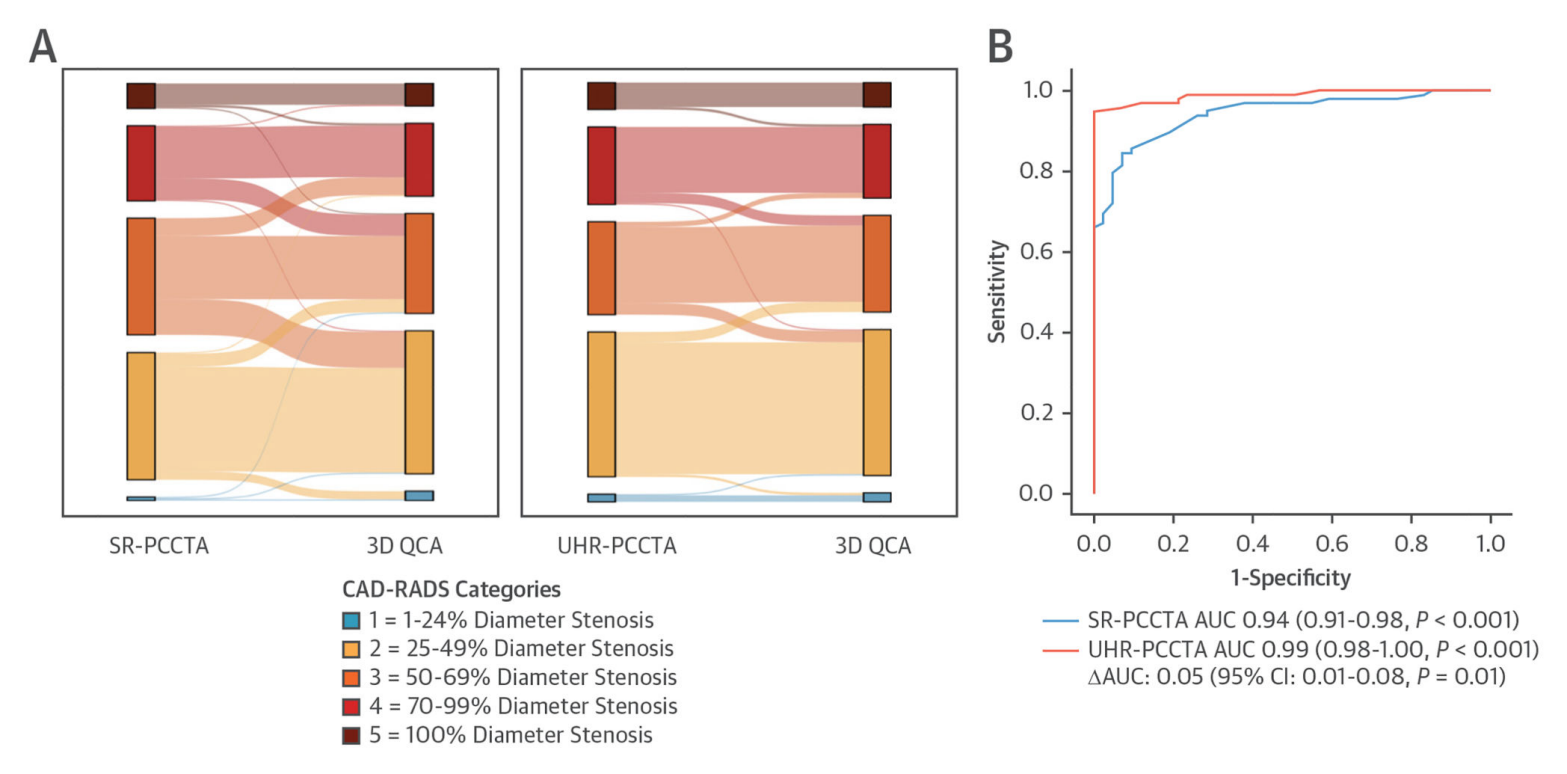
Diagnostic Classification According to SR-PCCTA and UHR-PCCTA Relative to 3D QCA (A) Sankey diagrams of plaque-level anatomic disease severity reclassification by SR-PCCTA and UHR-PCCTA relative to 3D QCA. (B) Diagnostic accuracy of UHR-PCCTA and SR-PCCTA in predicting coronary arteries with ≥50% diameter stenosis on 3D QCA. CAD-RADS = Coronary Artery Disease–Reporting and Data System; other abbreviations as in Figure 1.

Plaque-level anatomic disease severity classification according to SR- and UHR-PCCTA compared with 3D QCA shows reduced and less extensive misclassification with the use of UHR-PCCTA than with SR-PCCTA (Central Illustration C, Figure 5A). The per-plaque and per-vessel anatomic disease severity (according to CAD-RADS) agreement between UHR-PCCTA and 3D QCA was near-perfect across all stenoses, including severely calcific and nonseverely calcific stenoses (Table 6, Supplemental Table 2). There was substantial per-plaque agreement between UHR-PCCTA and 3D QCA in diffuse and focal stenoses, which improved to near-perfect on the per-vessel analysis. We observed a 7.6% vessel-level misclassification rate (4.1% overestimated and 3.5% underestimated).

Conversely, the per-plaque and per-vessel anatomic disease severity agreement between SR-PCCTA and 3D QCA was moderate across all severely calcific stenoses regardless of diffuse or focal pattern of disease (Table 6, Supplemental Table 2). This improved to substantial per-plaque and per-vessel agreement on a vessel-level analysis. We observed an 27.3% vessel-level misclassification rate (18.8% overestimated and 8.6% underestimated).

### Accuracy of PCCTA

The overall prevalence of coronary arteries with ≥50% diameter stenosis on 3D QCA was 70%. The diagnostic performance of SR-PCCTA and UHR-PCCTA for the identification of coronary arteries with ≥50% diameter stenosis on 3D QCA, expressed by the AUCs, was excellent: 0.94 (95% CI: 0.91-0.98; n = 170; *P* < 0.001) and 0.99 (95% CI: 0.98-1.00; n = 187; *P* < 0.01), respectively. Diagnostic accuracy results are presented in Table 7. In a head-to-head comparison of patients who had both UHR- and SR-PCCTA, the AUCs were significantly different: 0.05 (95% CI: 0.01-0.08; n = 139; *P* = 0.01) (Figure 5B). Diagnostic accuracy results are presented in Table 7.

In severely calcified vessels, the diagnostic performance of SR- and UHR-PCCTA remained excellent: AUCs: 0.92 (95% CI: 0.85-0.99; n = 61; *P* < 0.001) and 0.99 (95% CI: 0.96-1.00; n = 63; *P* < 0.001). The diagnostic performance of UHR-PCCTA was significantly better than that of SR-PCCTA: ΔAUC: 0.05 (95% CI: -0.03 to 0.12; n = 57; *P* = 0.01).

## Discussion

To the best of our knowledge, this is the first comparative evaluation of multienergy SR-PCCTA and UHR-PCCTA vs ICA in a series of patients with epicardial CAD presenting with ACS or referred for ICA. We first demonstrate that UHR-PCCTA provides outputs equivalent to ICA for the assessment of luminal stenosis, and its performance appears to be unaffected by coronary calcification, although it leads to significant increase of radiation exposure compared with SR-PCCTA. We then show that luminal evaluation with UHR-PCCTA is significantly more precise than SR-PCCTA evaluation. Third, we demonstrate that anatomic disease severity assessment is more accurate with UHR-PCCTA than with SR-PCCTA at a per-plaque and a per-vessel level regardless of calcification, with UHR-PCCTA outperforming SR-PCCTA for the identification of coronary arteries with diameter stenosis ≥50% on 3D QCA. These are clinically relevant findings, because UHR-PCCTA can now be used to define anatomically significant disease particularly in severely calcified arteries. Furthermore, we show that the existing digital subtraction algorithms applied to spectral data that enable digital removal of coronary calcification (Quantum PURE Lumen) are less precise for luminal evaluation than for monoenergetic image evaluation, leading to overestimation of the degree of stenosis. However, they can potentially be used as a tool to qualitatively rule out luminal stenosis.

This study is the first and largest evaluation of PCCTA imaging against the criterion standard of 3D QCA, including 100 patients with 343 plaques in 257 vessels. The results are promising for the evaluation of extensive and complex CAD with the use of SR-PCCTA imaging and particularly encouraging for UHR imaging. UHR-PCCTA has high precision, near-perfect agreement with 3D QCA for anatomic disease severity evaluation, and excellent diagnostic performance, particularly in heavily calcified and diffusely diseased vessels. Our findings extend the results of small case series showing high diagnostic accuracy of UHR-PCCTA for CAD detection in patients with severe calcific disease.^18^ In our subanalysis exploring the spectral capabilities of PCCTA for luminal evaluation of severely calcified plaques, analyzing virtual monoenergetic images at 100 keV or images postprocessed with the Quantum PURE Lumen algorithm did not improve luminal evaluation.

From a diagnostic perspective, UHR-PCCTA outperforms SR-PCCTA for luminal evaluation, anatomic disease severity assessment, and identification of disease requiring further pressure wire evaluation or treatment. These clinically relevant findings give further credence to earlier observations that UHR-PCCTA reduces the observed percentage diameter stenosis and reclassifies ~50% of patients with severe calcific CAD to a lower CAD-RADS category compared with lower-resolution reconstructions.^19^ Interestingly, the improvement in luminal evaluation with UHR-PCCTA is not confined to severely calcified arteries. Owing to the reduction of blooming artifact, it extends to diffuse diseased arteries, highlighting that it is beneficial for noninvasive imaging of patients with high burden of atherosclerotic disease. Therefore, because UHR-PCCTA imaging is more precise than SR-PCCTA, we speculate that the accuracy of functional disease severity evaluation by methods modeling flow with the use of coronary geometry (ieFFRCT) will improve with the use of UHR-PCCTA imaging. To date, early observations confined to SR-PCCTA support the equivalence of SR-PCCTA FFRCT with EID FFRCT.^20^ Ultimately, future dedicated studies may demonstrate that decision making with UHR-PCCTA will be similar to decision making with ICA.

Median radiation exposure from UHR imaging in our cohort was 7.5 mSv and 2.7-fold higher than SR-PCCTA imaging. In ROMICAT II (Multicenter Study to Rule Out Myocardial Infarction by Cardiac Computed Tomography; NCT01084239), coronary CTA participants had a mean radiation exposure of 11.3 mSv,^8^ whereas in RAPID CTCA (Rapid Assessment of Potential Ischaemic Heart Disease With CTCA; NCT02284191) and SYNTAX III Revolution (A Randomized Study Investigating the Use of CT Scan and Angiography of the Heart to Help the Doctors Decide Which Method Is the Best to Improve Blood Supply to the Heart in Patients With Complex Coronary Artery Disease; NCT02813473), conducted with later-generation scanners, median exposures were 5.8 and 5.0 mSv, respectively.^5,7^ Although UHR imaging leads to higher exposure than SR imaging, it is less than the mean radiation exposure of 10 ± 16 mSv from conventional diagnostic coronary angiography reported in SYNTAX III Revolution.

Taken together, in patients with high clinical likelihood of CAD in whom precise luminal evaluation is clinically important, UHR-PCCTA is superior to SR-PCCTA and can play a key role in future coronary CTA-first clinical management pathways. In the ACS context, the capabilities of UHR-PCCTA can be used to improve the diagnostic performance of coronary CTA and position it as a treatment decision making and revascularization planning noninvasive imaging modality. This new approach was piloted in the P3 study (Precise PCI Plan; NCT03782688)^17^ and is currently systematically evaluated with conventional EID coronary CTA in the P4 study (Precise Procedural and PCI Plan; NCT05253677). Ultimately, the combination of a treatment-centric coronary CTA approach with UHR-PCCTA imaging, as currently trialed in our unit, may satisfactorily address the drawbacks of the ROMICAT II and RAPID CTCA ACS studies.^5,7^

### Study Limitations

This study was delivered in a single center that is an early adopter of PCCTA imaging and recruited a small sample size, limiting the generalizability of the diagnostic accuracy findings, which should be further evaluated in larger dedicated multicenter studies. Enrollment of consecutive ACS participants was not feasible, because coronary CTA was available during week-day working hours only. Moreover, we were able to perform both SR-PCCTA and UHR-PCCTA in only 67 patients, whereas sex-based analyses were not performed because of the high proportion of male participants in the study, which is representative of the population distribution of CAD. Furthermore, although we include participants with previous coronary revascularization, we did not have sufficient participants to perform subanalyses dedicated to stented segments.

In addition, despite the collinearity in the quantitative assessment of the stenosis between PCCTA and 3D QCA, there was still a 15%-20% difference in stenosis degree assessment in Bland-Altman analysis. Notably, interobserver variability for 3D QCA analysis also is in the range of 15%-20%.^21^ In this context, it is worth highlighting that comparing absolute numbers between a 3D method (coronary CTA diameter stenosis is calculated as the circular equivalent at the site of maximum stenosis) relative to 3D QCA (which uses 3 planes to derive diameter stenosis) inherently introduces variability. Therefore, it is reasonable to continue reporting luminal stenosis in ranges, at least until validation relative to intravascular imaging is available. Finally, quantitative plaque assessment was not presented, because relevant tools for PCCTA are currently under development and require further validation.

## Conclusions

In patients presenting with ACS or referred for ICA, PCCTA luminal evaluation is strongly correlated with ICA 3D QCA luminal evaluation. UHR-PCCTA luminal evaluation is significantly more precise than SR-PCCTA, especially for heavily calcified lesions, and demonstrates excellent agreement with 3D QCA for anatomic disease severity evaluation, even when the multienergy internal subtraction capabilities of the SR-PCCTA are used. Although both UHR- and SR-PCCTA have excellent diagnostic performance, UHR imaging significantly outperforms SR-PCCTA for the identification of patients requiring further hemodynamic severity evaluation or revascularization (diameter stenosis ≥50% on 3D QCA), with a 2.7-fold increase in radiation exposure.

## Supplementary Material

Supplementary Material

## Figures and Tables

**Central Illustration:**
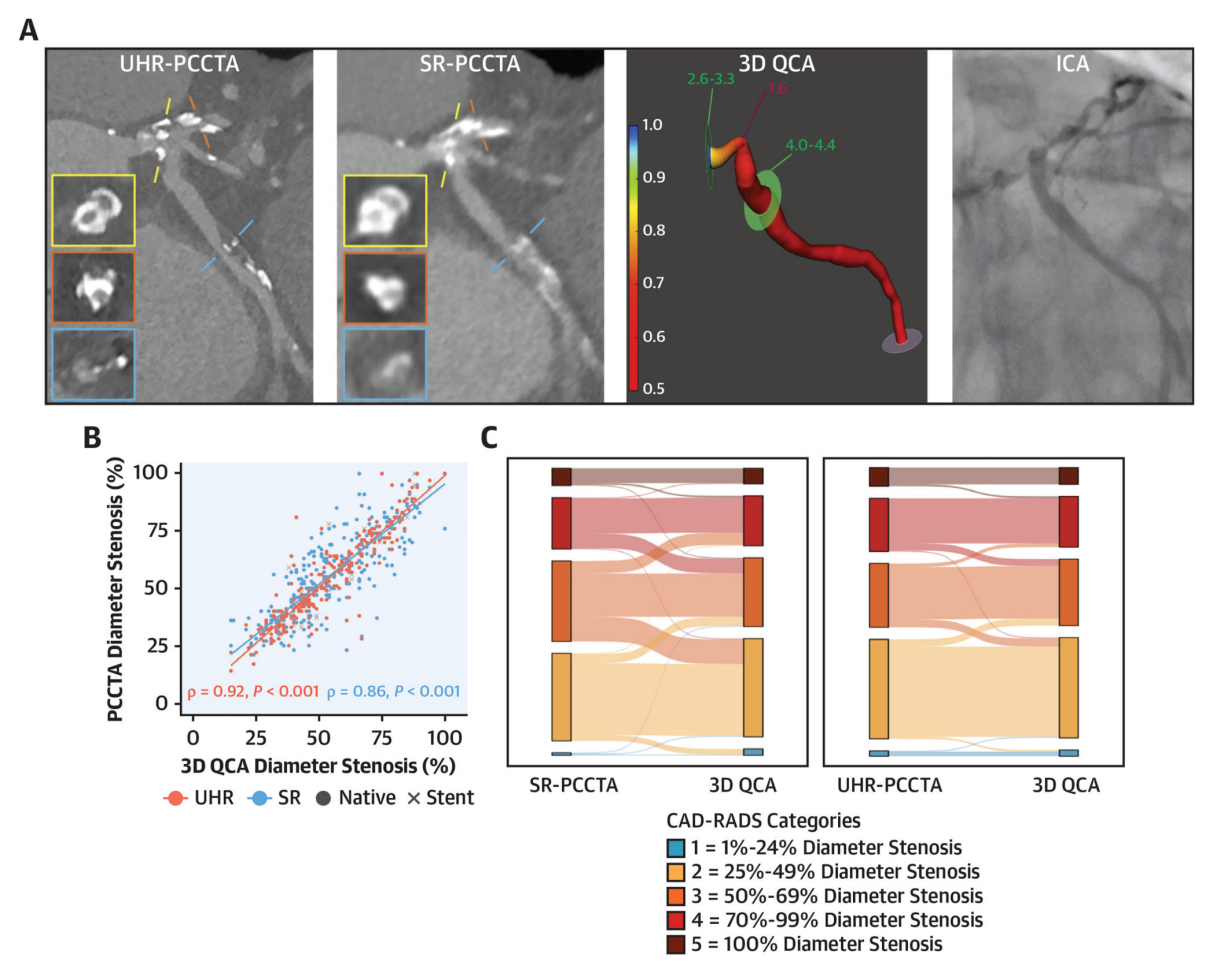
Comparison of Photon-Counting CT Angiography With Invasive Coronary Stenosis Assessment (A) Calcified plaques in the distal LM extending into the ostial LAD and the LCx causing severe stenoses, and a mixed plaque in the proximal LCx causing moderate stenosis (LM calcium score: 658 AU; LAD calcium score: 649 AU; LCx calcium score: 71 AU; patient calcium score: 1,533 AU). (B) Correlation of UHR- and SR-PCCTA diameter stenosis (%) relative to 3D QCA across all plaques, highlighting native and stented segments. (C) Sankey diagrams of plaque-level anatomical disease severity reclassification by SR-PCCTA and UHR-PCCTA relative to 3D QCA. 3D = 3-dimensional; CAD-RADS = Coronary Artery Disease– Reporting and Data System; ICA = invasive coronary angiography; LAD = left anterior descending artery; LCx = left circumflex artery; LM = left main coronary artery; PCCTA = photon-counting coronary computed tomographic angiography; QCA = quantitative coronary angiography; SR = standard resolution; UHR = ultrahigh resolution. Kotronias RA, et al. JACC Cardiovasc Imaging. 2025;18(5):572–585.

**Table 1:** Clinical and Imaging Characteristics (N = 100)

Clinical characteristics	
Age, y	64 (60-73)
Male	86 (86)
Hypertension	62 (62)
Hyperlipidemia	75 (75)
Diabetes	24 (24)
Smoker	47 (47)
Previous coronary artery disease	7(7)
Family history of premature IHD	19 (19)
≥2 risk factors	72 (72)
Non-STEACS presentation	61 (61)
STEACS presentation	13 (13)
Peak high-sensitivity troponin I, ng/L	1,376 (268-4,622)
PCCTA imaging	
Inpatient evaluation	74 (74)
Interval between ICA and PCCTA, d	1 (0-3)
SR-PCCTA alone	22 (22)
UHR-PCCTA alone	11 (11)
SR- and UHR-PCCTA	67 (67)
Systolic phase imaging	1 (1)
Flex UHR-PCCTA	68 (68)
Spiral UHR-PCCTA	10 (10)
Total coronary calcium score, AU	391 (52-962)

Values are median (Q1-Q3) or n (%).

ACS = acute coronary syndrome; AU = arbitrary units; ICA = invasive coronary angiography; IHD = ischemic heart disease; PCCTA = photon-counting coronary computed tomographic angiography; SR = standard resolution; STEACS = ST-segment elevation acute coronary syndrome; UHR = ultrahigh resolution.

**Table 2:** Vessel Characteristics

Total number of epicardial coronary arteries included	257
Number of arteries/patient with ≥1 visible plaque	2 (1-3)
Left main stem	9 (4)
Left anterior descending artery	77 (30)
Left circumflex artery	49 (19)
Right coronary artery	69 (27)
Intermediate	4 (1)
Diagonal	23 (9)
Obtuse marginal	26 (10)
Total number of coronary lesions included	343
Lesions per patient	3 (2-5)
Lesions according to calcification severity^a^	
Severe (CCS >300 AU)	124 (38)
Moderate (CCS100-300 AU)	67 (20)
Mild (CCS 0-100 AU)	69 (21)
None	71 (21)
Lesions in diffusely diseased arteries (QFR PPG <0.66)^b^	114 (35)
Lesions in stents	12 (4)

Values are median (Q1-Q3) or n (%).

a Out of 331 (excluding stented segments).

b Out of 322 (excluding vessels where QFR PPG not evaluable).

CAD = coronary artery disease; CCS = coronary calcium score; QFR-PPG = quantitative flow reserve–based pressure pull back gradient; other abbreviations as in Table 1.

**Table 3:** Plaque Characteristics and Stenosis Quantification

	3D QCA	SR-PCCTA	UHR-PCCTA
	(n = 343)	(n = 292)	(n = 271)
Characteristics			
% diameter stenosis	53 (40-71)	56 (40-72)	54 (39-72)
Minimum luminal diameter, mm	1.1 (0.7-1.6)	1.2 (0.7-1.7)	1.1 (0.6-1.50)
CAD-RADS			
1	8 (2)	3 (1)	6 (2)
2	143 (42)	107 (36)	112 (41)
3	98 (29)	98 (34)	72 (27)
4	71 (21)	63 (22)	60 (22)
5	23(7)	21 (7)	21 (8)

Values are median (Q1-Q3) or n (%).

3D = 3-dimensional; CAD-RADS = Coronary Artery Disease–Reporting and Data System; QCA = quantitative coronary angiography; other abbreviations as in Table 1.

**Table 4:** Comparative Evaluation of PCCTA Relative to 3D QCA

	3D QCA vs SR-PCCTA			3D QCA vs UHR-PCCTA			SR-PCCTA vs UHR-PCCTA	
	Correlation, ρ	ICC		Correlation, ρ	ICC		Correlation, ρ	ICC
All stenoses	n = 292			n = 271			n = 228	
% diameter stenosis	0.86 (0.82-0.89)	0.87 (0.84-0.90)		0.92 (0.90-0.94)	0.94 (0.92-0.95)		0.86 (0.82-0.89)	0.87 (0.83-0.90)
MLD	0.83 (0.79-0.86)	0.84 (0.80-0.87)		0.90 (0.87-0.92)	0.90 (0.88-0.92)		0.85 (0.81-0.89)	0.87 (0.83-0.90)
Severely calcific stenoses	n = 101			n = 108			n = 86	
% diameter stenosis	0.84 (0.77-0.89)	0.86 (0.80-0.90)		0.89 (0.84-0.92)	0.91 (0.87-0.94)		0.88 (0.81-0.92)	0.89 (0.83-0.93)
MLD	0.85 (0.78-0.90)	0.83 (0.76-0.88)		0.87 (0.81-0.91)	0.88 (0.83-0.92)		0.85 (0.77-0.90)	0.85 (0.77-0.90)
Nonseverely calcific stenoses	n = 185			n = 151			n = 136	
% diameter stenosis	0.87 (0.83-0.90)	0.88 (0.84-0.91)		0.95 (0.94-0.97)	0.96 (0.95-0.97)		0.86 (0.81-0.90)	0.87 (0.82-0.90)
MLD	0.84 (0.78-0.88)	0.86 (0.82-0.89)		0.92 (0.89-0.94)	0.92 (0.89-0.94)		0.87 (0.82-0.90)	0.89 (0.85-0.92)
Diffuse pattern	n = 91			n = 98			n = 75	
% diameter stenosis	0.80 (0.71-0.87)	0.81 (0.72-0.87)		0.89 (0.84-0.93)	0.89 (0.84-0.93)		0.83 (0.75-0.89)	0.84 (0.76-0.90)
MLD	0.68 (0.55-0.78)	0.74 (0.64-0.82)		0.81 (0.73-0.87)	0.85 (0.78-0.90)		0.74 (0.62-0.83)	0.86 (0.79-0.91)
Focal pattern	n = 181			n = 157			n = 134	
% diameter stenosis	0.83 (0.78-0.87)	0.84 (0.79-0.88)		0.92 (0.89-0.94)	0.93 (0.91-0.95)		0.82 (0.75-0.87)	0.83 (0.77-0.88)
MLD	0.83 (0.78-0.87)	0.83 (0.77-0.87)		0.90 (0.86-0.92)	0.89 (0.85-0.92)		0.84 (0.78-0.89)	0.83 (0.77-0.88)

Values are HR (95% CI), unless otherwise indicated. All correlations and agreements are significant (*P* < 0.001). ICC = intraclass correlation; MLD = minimum luminal diameter; other abbreviations as in Table 1.

**Table 5:** Comparative Evaluation of SR-PCCTA and UHR-PCCTA Relative to 3D QCA for Severely Calcific Plaques

	3D QCA vs SR-PCCTA 67 keV			3D QCA vs SR-PCCTA 100 keV			3D QCA vs SR-PCCTA Quantum PURE Lumen			3D QCA vs UHR-PCCTA	
	Correlation, ρ	ICC		Correlation, ρ	ICC		Correlation, ρ	ICC		Correlation, ρ	ICC
% diameter stenosis	0.82 (0.73-0.89)	0.86 (0.78-0.91)		0.78 (0.67-0.85)	0.77 (0.66-0.84)		0.63 (0.47-0.75)	0.61 (0.44-0.73)		0.86 (0.78-0.91)	0. 90 (0.85-0.93)
MLD	0.85 (0.77-0.90)	0.83(0.75-0.89)		0.72 (0.59-0.81)	0.71 (0.58-0.81)		0.75 (0.63-0.84)	0.74 (0.62-0.83)		0.86 (0.79-0.91)	0.87 (0.80-0.91)

Values are HR (95% CI), unless otherwise indicated. All correlations and agreements are significant (*P* < 0.001). Abbreviations as in Tables 1, 3, and 4.

**Table 6:** Comparative Evaluation of PCCTA Anatomic Disease Severity Classification Relative to 3D QCA

	3D QCA vs SR-PCCTA	3D QCA vs UHR-PCCTA
ALL		
Per-plaque CAD-RADS	0.57 (0.49-0.64); n = 292	0.82 (0.76-0.87); n = 271
Per-vessel CAD-RADS	0.63 (0.54-0.71); n = 187	0.90 (0.84-0.95); n = 170
Severely calcific vessels		
Per-plaque CAD-RADS	0.51 (0.39-0.64); n = 101	0.82 (0.72-0.90); n = 108
Per-vessel CAD-RADS	0.54 (0.37-0.70); n = 61	0.89 (0.79-0.98); n = 63
Nonseverely calcific vessels		
Per-plaque CAD-RADS	0.61 (0.51-0.70); n = 185	0.82 (0.74-0.89); n = 151
Per-vessel CAD-RADS	0.67 (0.56-0.77); n = 124	0.90 (0.83-0.97); n = 107
Diffuse pattern		
Per-plaque CAD-RADS	0.57 (0.42-0.70); n = 91	0.78 (0.66-0.88); n = 98
Per-vessel CAD-RADS	0.63 (0.40-0.82); n = 46	0.88 (0.75-0.99); n = 50
Focal pattern		
Per-plaque CAD-RADS	0.51 (0.41-0.61); n = 181	0.80 (0.72-0.88); n = 157
Per-vessel CAD-RADS	0.56 (0.44-0.67); n = 124	0.88 (0.79-0.95); n = 107

Values are Cohen’s κ (95% CI). All agreements are significant *P* < 0.001. Abbreviations as in Tables 1 and 3.

**Table 7:** Diagnostic Accuracy of SR-PCCTA and UHR-PCCTA for the Identification of Coronary Arteries With ≥50% Luminal Stenosis on 3D QCA

	All Coronary Arteries			Coronary Arteries Imaged With UHR- and SR-PCCTA	
	SR-PCCTA (n = 187)	UHR-PCCTA (n = 170)		SR-PCCTA (n = 139)	UHR-PCCTA (n = 139)
Sensitivity, %	87	96		85	95
Specificity, %	88	100		93	100
Positive likelihood ratio	7.25	Certainty		12	Certainty
Negative likelihood ratio	0.14	0.04		0.16	0.05

The optimum cutoff diameter stenoses were determined according to the Youden index and found to be 55% for SR-PCCTA and 51% for UHR-PCCTA.

Abbreviations as in Tables 1 and 3.

## Perspectives

### Competency in practice-based learning

PCCTA luminal evaluation compares favorably with invasive quantitative coronary angiography. UHR-PCCTA luminal evaluation is more precise than standard-resolution PCCTA, especially in heavily calcified lesions. UHR-PCCTA diagnostically outperforms standard-resolution PCCTA.

### Translational outlook

UHR-PCCTA imaging overcomes several of the common drawbacks of contemporary CT and may in the future be used for treatment decision making and revascularization planning.

## Abbreviations and Acronyms

3D: 3-dimensional
ACS: acute coronary syndrome
CAD: coronary artery disease
CTA: computed tomographic angiography
EID: energy integrating detector
ICA: invasive coronary angiography
NSTEACS: non–ST-segment elevation acute coronary syndrome
PCCTA: photon-counting computed tomographic angiography
QCA: quantitative coronary angiography
QFR-PPG: quantitative flow reserve–based pressure pull back gradient
SR: standard resolution
UHR: ultrahigh resolution
