# Supplementary material for: Benchmarking Photon-Counting Computed Tomography Angiography Against Invasive Assessment of Coronary Stenosis: Implications for Severely Calcified Coronaries
Source: JACC Cardiovasc Imaging. Author manuscript; Available in PMC 2025 Dec 1. (PMC7618422; doi:10.1016/j.jcmg.2024.11.005)
Supplement: Supplementary Material [file EMS210953-supplement-Supplementary_Material.zip › 1-s2.0-S1936878X25000269-mmc2.docx]

| **Extended OxAMI & ORFAN Study Collaborators list** | | | |
| --- | --- | --- | --- |
| **First Name and Middle Initial(s)** | **Last Name** | Institution | Location |
| Robin | Choudhury | Division of Cardiovascular Medicine, Radcliffe Department of Medicine, University of Oxford | Oxford, UK |
| Sam | Dawkins | Oxford University Hospitals NHS Trust | Oxford UK |
| Wendy | Qin | ORFAN core laboratory, Acute Multidisciplinary Imaging and Interventional Centre, Division of Cardiovascular Medicine, Radcliffe Department of Medicine, University of Oxford | Oxford, UK |
| Mary | Kasongo | ORFAN core laboratory, Acute Multidisciplinary Imaging and Interventional Centre, Division of Cardiovascular Medicine, Radcliffe Department of Medicine, University of Oxford | Oxford, UK |
| Chrisha | Ledesma | ORFAN core laboratory, Acute Multidisciplinary Imaging and Interventional Centre, Division of Cardiovascular Medicine, Radcliffe Department of Medicine, University of Oxford | Oxford, UK |
| Damaris | Darby | ORFAN core laboratory, Acute Multidisciplinary Imaging and Interventional Centre, Division of Cardiovascular Medicine, Radcliffe Department of Medicine, University of Oxford | Oxford, UK |
| Bruno | Silva Santos | ORFAN core laboratory, Acute Multidisciplinary Imaging and Interventional Centre, Division of Cardiovascular Medicine, Radcliffe Department of Medicine, University of Oxford | Oxford, UK |
| Alexios S | Antonopoulos | Acute Multidisciplinary Imaging and Interventional Centre, Division of Cardiovascular Medicine, Radcliffe Department of Medicine, University of Oxford | Oxford, UK |
| Michail C | Mavrogiannis | ORFAN core laboratory, Acute Multidisciplinary Imaging and Interventional Centre, Division of Cardiovascular Medicine, Radcliffe Department of Medicine, University of Oxford | Oxford, UK |
| Andrew | Kelion | Oxford University Hospitals NHS Foundation Trust | Oxford, UK |
| Susan | Anthony | Oxford University Hospitals NHS Foundation Trust | Oxford, UK |
| Adrian | Banning | Oxford University Hospitals NHS Foundation Trust | Oxford, UK |
| Lucy | Kingham | Division of Cardiovascular Medicine, Radcliffe Department of Medicine, University of Oxford | Oxford, UK |
| Rajesh K | Kharbanda | Division of Cardiovascular Medicine, Radcliffe Department of Medicine, University of Oxford | Oxford, UK |
| Chris | Mathers | Caristo Diagnostics | Oxford, UK |
| Tarun K | Mittal | Royal Brompton and Harefield Hospitals | London, UK |
| Anne | Rose | Milton Keynes University Hospital NHS Foundation Trust | Milton Keynes, UK |
| George | Hudson | Department of Cardiovascular Sciences and National Institute of Health Research Leicester Biomedical Research Centre, University of Leicester | Leicester, UK |
| Amrita | Bajaj | Department of Cardiovascular Sciences and National Institute of Health Research Leicester Biomedical Research Centre, University of Leicester | Leicester, UK |
| Intrajeet | Das | Department of Cardiovascular Sciences and National Institute of Health Research Leicester Biomedical Research Centre, University of Leicester | Leicester, UK |
| Aparna | Deshpande | Department of Cardiovascular Sciences and National Institute of Health Research Leicester Biomedical Research Centre, University of Leicester | Leicester, UK |
| Praveen | Rao | Department of Cardiovascular Sciences and National Institute of Health Research Leicester Biomedical Research Centre, University of Leicester | Leicester, UK |
| Dan | Lawday | Department of Cardiovascular Sciences and National Institute of Health Research Leicester Biomedical Research Centre, University of Leicester | Leicester, UK |
| Francesca | Pugliese | Barts Heart Centre, St Bartholomew's Hospital, Barts Health NHS Trust; William Harvey Research Institute, Barts and The London School of Medicine and Dentistry, Queen Mary University of London | London, UK |
| Steffen E | Petersen | Barts Heart Centre, St Bartholomew's Hospital, Barts Health NHS Trust; William Harvey Research Institute, Barts and The London School of Medicine and Dentistry, Queen Mary University of London | London, UK |
| Saeed | Mirsadraee | Royal Brompton and Harefield NHS Foundation Trust | London, UK |
| Nicholas | Screaton | Royal Papworth Hospital | Cambridge, UK |
| Jonathan | Rodrigues | Royal United Hospitals Bath NHS Foundation Trust and Department of Health, University of Bath, | Bath, UK |
| Benjamin | Hudson | Royal United Hospitals Bath NHS Foundation Trust and Department of Health, University of Bath, | Bath, UK |
| John | Graby | Royal United Hospitals Bath NHS Foundation Trust and Department of Health, University of Bath, | Bath, UK |
| Colin | Berry | NHS Greater Glasgow and Clyde | Glasgow, UK |
| Mohamed | Marwan | Department of Cardiology, Friedrich-Alexander-Universität Erlangen-Nürnberg | Erlangen, Germany |
| Pál | Maurovich-Horvat | Department of Radiology, MTA-SE Cardiovascular Imaging Research Group | Budapest, Hungary |
| Guo-Wei | He | TEDA International Cardiovascular Hospital | Tianjin, China |
| Wen-Hua | Lin | TEDA International Cardiovascular Hospital | Tianjin, China |
| Li-Juan | Fan | TEDA International Cardiovascular Hospital | Tianjin, China |
| Naohiko | Takahashi | Oita University | Japan |
| Hidekazu | Kondo | Oita University | Japan |
| Neng | Dai | Fudan University | Shanghai, China |
| Junbo | Ge | Fudan University | Shanghai, China |
| Bon-Kwon | Koo | Seoul National University | Seoul, South Korea |
| Marco | Guglielmo | Centro Cardiologico Monzino IRCCS, University of Milan | Milan, Italy |
| Gianluca | Pontone | Centro Cardiologico Monzino IRCCS, University of Milan | Milan, Italy |
| Daniel | Huck | Brigham and Women’s Hospital | Boston, USA |
| Theodora | Benedek | University of Medicine and Pharmacy of Tirgu Mures | Romania |
| Ronak | Rajani | Guy’s and St Thomas’ NHS Foundation Trust | London, UK |
| Dijana | Vilic | Guy’s and St Thomas’ NHS Foundation Trust | London, UK |
| Haleema | Aljazzaf | Guy’s and St Thomas’ NHS Foundation Trust | London, UK |
| Mak S | Mun | Guy’s and St Thomas’ NHS Foundation Trust | London, UK |
| Giulia | Benedetti | Guy’s and St Thomas’ NHS Foundation Trust | London, UK |
| Rebecca L | Preston | Guy’s and St Thomas’ NHS Foundation Trust | London, UK |
| Zahra | Raisi-Estabragh | Queen Mary University of London | London, UK |
| Derek L | Connolly | Sandwell and West Birmingham Hospitals NHS Trust | Birmingham, UK |
| Vinoda | Sharma | Sandwell and West Birmingham Hospitals NHS Trust | Birmingham, UK |
| Rebecca | Grenfell | Sandwell and West Birmingham Hospitals NHS Trust | Birmingham, UK |
| William | Bradlow | University Hospitals Birmingham (UHB) NHS Trust | Birmingham, UK |
| Matthias | Schmitt | University Hospital of Manchester Foundation Trust | Manchester, UK |
| Fabiano | Serfaty | Serfaty Clinicas, Rio de Janeiro | Brazil |
| Ilan | Gottlieb | Casa de Saúde São José, Rio de Janeiro | Brazil |
| Mario FT | Neves | Universidade do Estado do Rio de Janeiro | Rio de Janeiro, Brazil |
| David E | Newby | University of Edinburgh | Edinburgh, UK |
| Marc R | Dweck | University of Edinburgh | Edinburgh, UK |
| Bernard J | Gersh | Cleveland Clinic Heart and Vascular Institute, Cleveland | OH, USA |
| Stéphane | Hatem | Foundation for Innovation in Cardiometabolism and Nutrition (ICAN) | Paris, France |
| Alban | Redheuil | Sorbonne Université, Faculté de Médecine Pierre et Marie Curie | Paris, France |
| Georgios | Benetos | Lefkos Stavros Clinic Athens | Greece |
| Meinrad | Beer | Universitätsklinikum Ulm | Germany |
| Gastón A | Rodriguez-Granillo | ENERI Medical Institute | Argentina |
| Joseph | Selvanayagam | South Australia Health and Medical Research Institute | Adelaide, Australia |
| Francisco | Lopez-Jimenez | Mayo Clinic | Rochester, USA |
| Ruben | De Bosscher | University Hospitals Leuven | Belgium |
| Alain | Tavildari | Cardiovista | France |
| Gemma | Figtree | University of Sydney | Australia |
| Ibrahim | Danad | Amsterdam University Medical Centers | Netherlands |
| Ronney | Shantouf | Cleveland Clinic Abu Dhabi | UAE |
| Bas | Kietselaer | Zuyderland Medical Centre | Heerlen, Netherlands |
| Dimitris | Tousoulis | University of Athens | Greece |
| George | Dangas | Mount Sinai School of Medicine | New York, USA |
| Nehal N | Mehta | National Institutes of Health, National Heart, Lung, and Blood Institute | Bethesda, Maryland, USA |
| Christos | Kotanidis | Newcastle Upon Tyne Hospitals NHS Foundation Trust | Newcastle, UK |
| Vijay | Kunadian | Newcastle Upon Tyne Hospitals NHS Foundation Trust | Newcastle, UK |
| Timothy A | Fairbairn | Liverpool Heart and Chest Hospital NHS Foundation Trust | Liverpool, UK |
