# Supplementary material for: Benchmarking Photon-Counting Computed Tomography Angiography Against Invasive Assessment of Coronary Stenosis: Implications for Severely Calcified Coronaries
Source: JACC Cardiovasc Imaging. Author manuscript; Available in PMC 2025 Dec 1. (PMC7618422; doi:10.1016/j.jcmg.2024.11.005)
Supplement: Supplementary Material [file EMS210953-supplement-Supplementary_Material.zip › 1-s2.0-S1936878X25000269-mmc1.docx]

**Supplemental Methods**

**Detailed breakdown of reasons for nonassessable segments**

We assessed 2490 segments and 120 were not assessable. Briefly, 113 segments were not assessable as the vessel was occluded proximal to the segment, precluding reliable luminal assessment on CCTA. Three segments (AHA 1, 11, 13) from one UHR scan were not assessable due to motion artefact (during the acquisition, the participant went into complete heart block in the context of a large inferior STEMI) which introduced significant motion artefact. The same participant had a fully assessable SR-PCCTA. In another participant, one segment (AHA 10) from an UHR scan was not assessable due to stitch artefact (Flex protocol and significant variation in RR interval). This segment was assessable on the SR-PCCTA. In another patient, 3 segments from an SR PCCTA were not assessable as the participant significantly changed their breathing following planning resulting to AHA segments 1, 5, and 6 being out of the acquisition field. The UHR scan that was adjusted for the breathing was fully assessable. These segments were not included in the head-to-head comparison.

**Supplemental Table 1 – CAD-RADS Grading Scale for Stenosis Severity**

| **CAD-RADS Grading Scale for Stenosis Severity** | **Degree of luminal stenosis** |
| --- | --- |
| 0 | No visible stenosis |
| 1 | 1-24% |
| 2 | 25-49% |
| 3 | 50-69% |
| 4 | 70-99% or left main ≥50% |
| 5 | Occluded |

*CAD-RADS: Coronary Artery Disease - Reporting and Data System*

**Supplemental Table 2 – Anatomic Disease Classification Agreement**

| **3D QCA CAD-RADS** | **SR-PCCTA** | **UHR-PCCTA** |
| --- | --- | --- |
| **Per-plaque analysis according to CAD-RADS stenosis categorisation** | | |
| 1 | 1/8 (13%) | 5/7 (71%) |
| 2 | 88/120 (73%) | 102/113 (90%) |
| 3 | 43/84 (51%) | 59/75 (79%) |
| 4 | 43/61 (70%) | 51/57 (89%) |
| 5 | 18/19 (95%) | 19/19 (100%) |
| **Per-vessel analysis according to CAD-RADS stenosis categorisation** | | |
| 1 | 1/5 (20%) | 4/4 (100%) |
| 2 | 39/54 (72%) | 45/48 (94%) |
| 3 | 36/55 (65%) | 42/49 (86%) |
| 4 | 42/54 (78%) | 47/50 (94%) |
| 5 | 18/19 (95%) | 19/19 (100%) |

*CAD-RADS: Coronary Artery Disease - Reporting and Data System; PCCTA: photon-counting coronary computed tomography angiography; SR: standard resolution; UHR: ultrahigh resolution; 3D QCA: 3-dimensional quantitative coronary angiography*


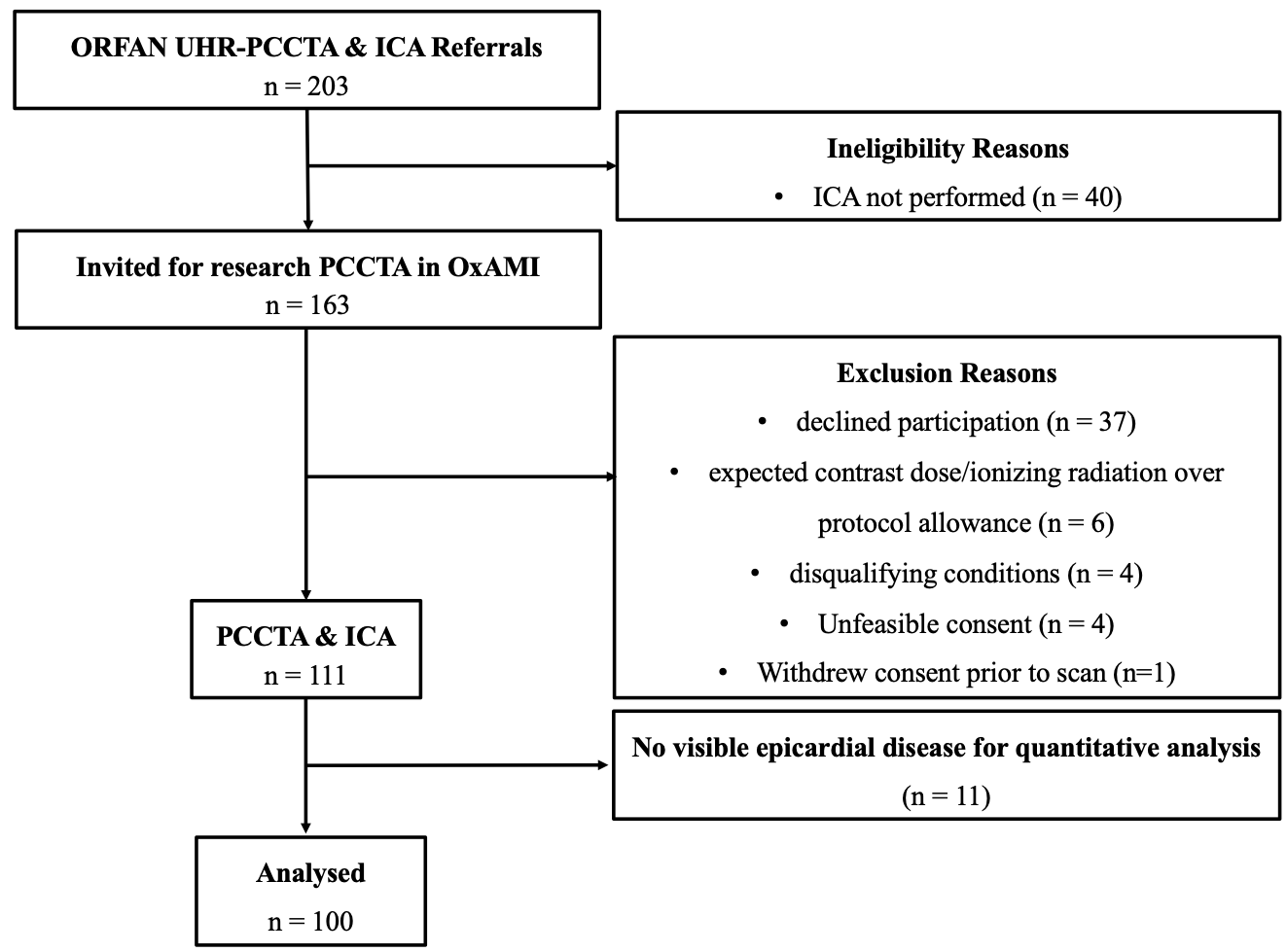


**Supplemental Figure 1. Study Flow Diagram**

*ICA: invasive coronary angiography; PCCTA: photon-counting coronary computed tomography angiography; UHR: ultrahigh resolution*


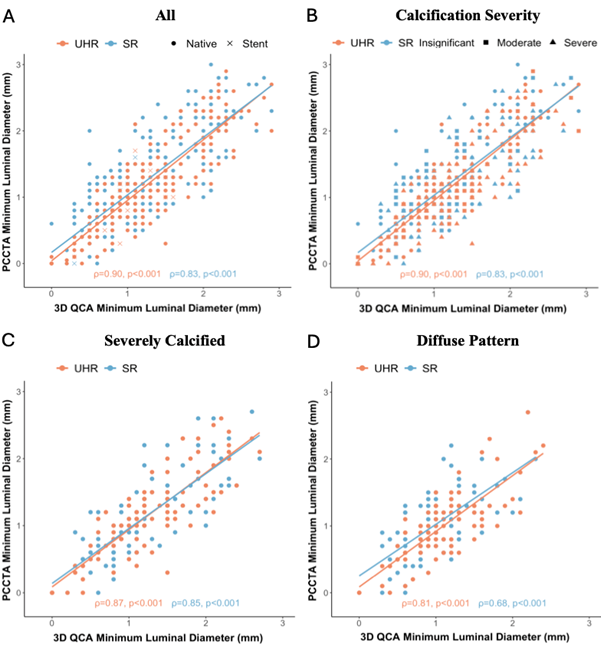


**Supplemental Figure 2. Correlations of UHR- and SR-PCCTA minimum luminal diameter (mm) relative to 3D QCA.** (A) Across all plaques highlighting native and stented segments; (B) across all plaques highlighting different degrees of calcification; (C) plaques in severely calcified plaques; (D) plaques in diffusely diseased arteries.

*PCCTA: photon-counting coronary computed tomography angiography; SR: standard resolution;* *UHR: ultrahigh resolution; 3D QCA: 3-dimensional quantitative coronary angiography*


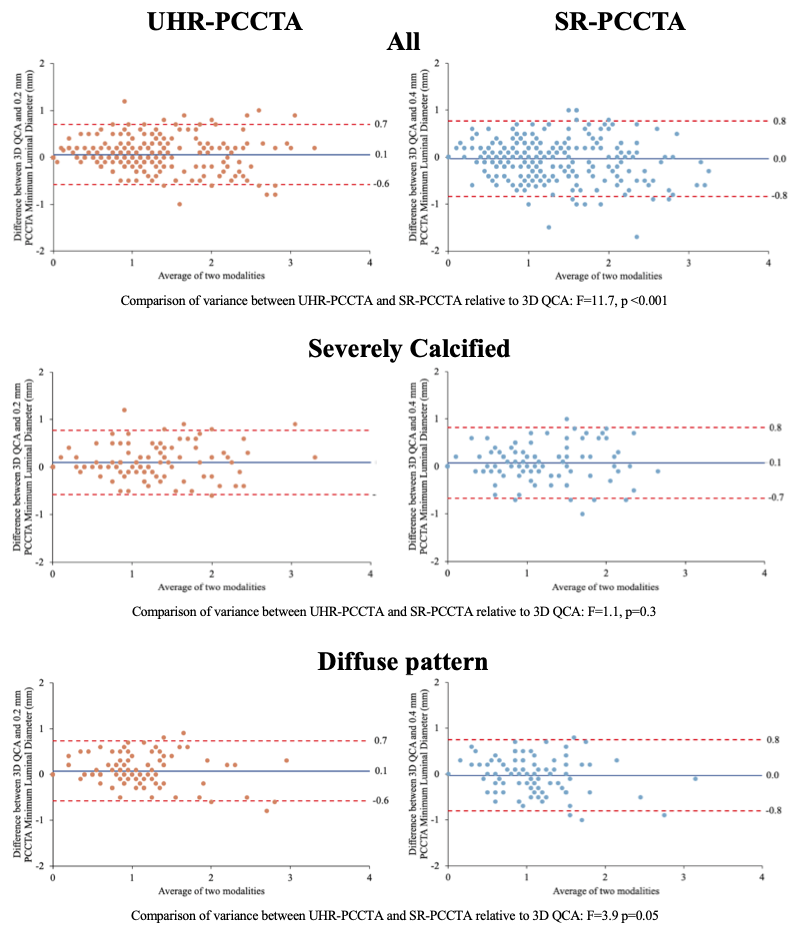


**Supplemental Figure 3.** **Bland-Altman plots of UHR-PCCTA and SR-PCCTA minimum luminal diameter (mm) relative to 3D QCA for all plaques, severely calcified plaques, and plaques in diffusely diseased vessels.**

*PCCTA: photon-counting coronary computed tomography angiography; SR: standard resolution;* *UHR: ultrahigh resolution; 3D QCA: 3-dimensional quantitative coronary angiography*


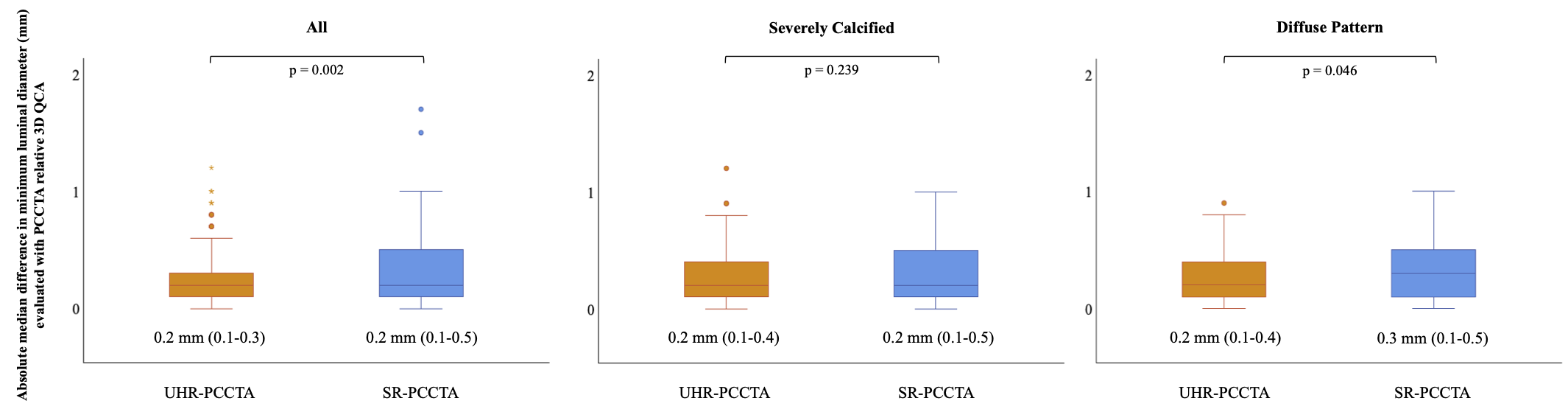


**Supplemental Figure 4. Box plot of the absolute mean difference in minimum luminal diameter (mm) evaluation of UHR-PCCTA and SR-PCCTA relative to 3D QCA.**  **Values are presented as median (IQR).**

*PCCTA: photon-counting coronary computed tomography angiography; SR: standard resolution;* *UHR: ultrahigh resolution; 3D QCA: 3-dimensional quantitative coronary angiography*


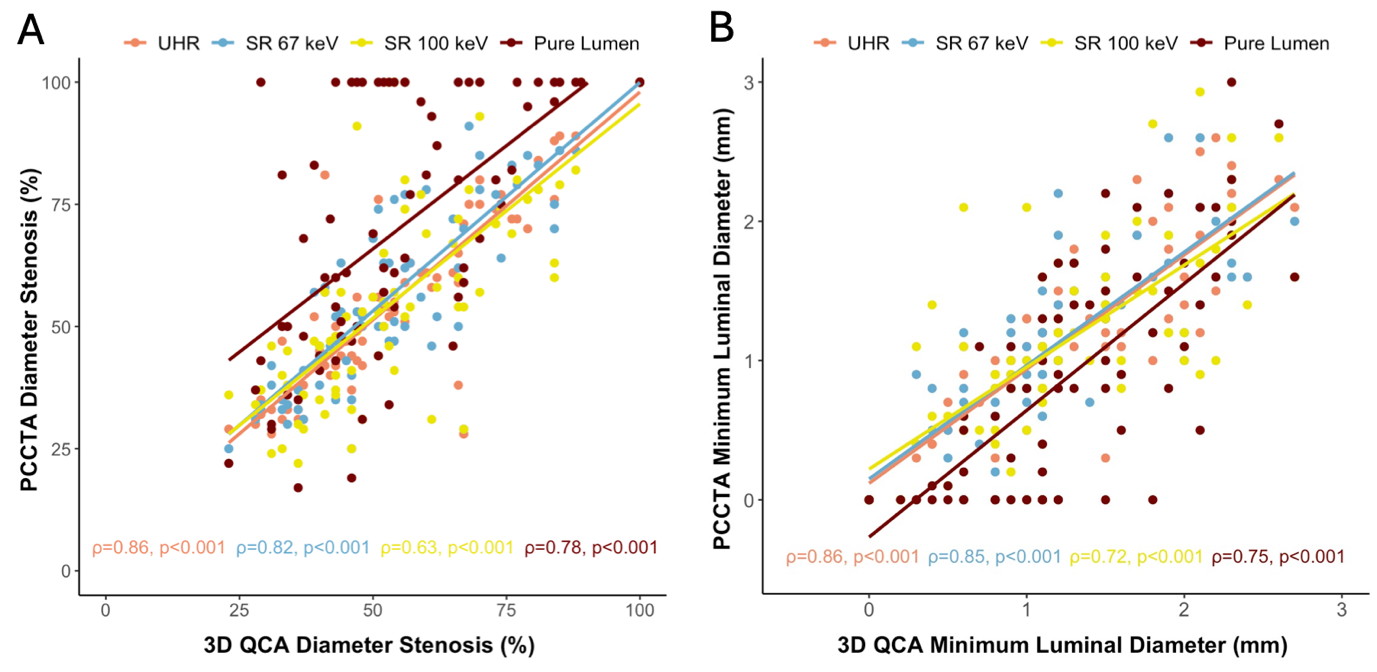


**Supplemental Figure 5. Correlations of UHR, SR-PCCTA read at 67 keV, 100 keV and SR-PCCTA (A) Quantum PURE Lumen diameter stenosis (%) and (B) minimum luminal diameter (mm) relative to 3D QCA in severely calcified arteries (>300 AU).**

*AU: Agatston units; PCCTA: photon-counting coronary computed tomography angiography; SR: standard resolution;* *UHR: ultrahigh resolution; 3D QCA: 3-dimensional quantitative coronary angiography*
